# Differential Activation of *TAS2R4* May Recover Ability to Taste Propylthiouracil for Some *TAS2R38* AVI Homozygotes

**DOI:** 10.3390/nu16091357

**Published:** 2024-04-30

**Authors:** Alissa A. Nolden, Maik Behrens, John E. McGeary, Wolfgang Meyerhof, John E. Hayes

**Affiliations:** 1 Department of Food Science, University of Massachusetts, Amherst, MA 01003, USA; anolden@umass.edu; 2 Sensory Evaluation Center, The Pennsylvania State University, University Park, PA 16802, USA; 3 Department of Food Science, The Pennsylvania State University, University Park, PA 16802, USA; 4 German Institute of Human Nutrition Potsdam-Rehbruecke, Department Molecular Genetics, 14558 Nuthetal, Germany; m.behrens.leibniz-lsb@tum.de (M.B.); wolfgang.meyerhof@uks.eu (W.M.); 5 Leibniz Institute for Food Systems Biology, Technical University of Munich, 85354 Freising, Germany; 6 Center of Innovation in Long Term Services & Supports, VA Providence Healthcare, Providence, RI 02908, USA; john.mcgeary@brown.edu; 7 Department of Psychiatry & Human Behavior, Alpert Medical School of Brown University, Providence, RI 02903, USA; 8 Center for Integrative Physiology and Molecular Medicine, Saarland University, 66424 Homburg, Germany

**Keywords:** propylthiouracil, phenylthiocarbamide, individual differences, supertasting, suprathreshold, psychophysics, genetic variation

## Abstract

Bitterness from phenylthiocarbamide and 6-n-propylthiouracil (PROP) varies with polymorphisms in the *TAS2R38* gene. Three SNPs form two common (AVI, PAV) and four rare haplotypes (AAI, AAV, PVI, and PAI). AVI homozygotes exhibit higher detection thresholds and lower suprathreshold bitterness for PROP compared to PAV homozygotes and heterozygotes, and these differences may influence alcohol and vegetable intake. Within a diplotype, substantial variation in suprathreshold bitterness persists, and some AVI homozygotes report moderate bitterness at high concentrations. A second receptor encoded by a gene containing a functional polymorphism may explain this. Early work has suggested that PROP might activate TAS2R4 in vitro, but later work did not replicate this. Here, we identify three *TAS2R4* SNPs that result in three diplotypes—SLN/SLN, FVS/SLN, and FVS/FVS—which make up 25.1%, 44.9%, and 23.9% of our sample. These *TAS2R4* haplotypes show minimal linkage disequilibrium with *TAS2R38,* so we examined the suprathreshold bitterness as a function of both. The participants (*n* = 243) rated five PROP concentrations in duplicate, interleaved with other stimuli. As expected, the *TAS2R38* haplotypes explained ~29% (*p* < 0.0001) of the variation in the bitterness ratings, with substantial variation within the haplotypes (AVI/AVI, PAV/AVI, and PAV/PAV). Notably, the *TAS2R4* diplotypes (independent of the *TAS2R38* haplotypes) explained ~7–8% of the variation in the bitterness ratings (*p* = 0.0001). Given this, we revisited if PROP could activate heterologously expressed TAS2R4 in HEK293T cells, and calcium imaging indicated 3 mM PROP is a weak TAS2R4 agonist. In sum, our data are consistent with the second receptor hypothesis and may explain the recovery of the PROP tasting phenotype in some AVI homozygotes; further, this finding may potentially help explain the conflicting results on the *TAS2R38* diplotype and food intake.

## 1. Introduction

Bitterness is classically framed as an evolutionary mechanism to defend against the ingestion of toxins found in plants [1,2,3], with the caveat that many bitter stimuli are not toxic [4,5]. To detect a wide range of structurally diverse bitter compounds, humans have numerous bitter taste receptors (TAS2Rs) encoded by ~26 functional genes (*TAS2Rs*) [6,7]. Given modern food safety regulatory regimes, we no longer rely on this defense mechanism for survival, and the view that bitter equals bad is an oversimplification [8,9]. Many compounds in the human diet elicit bitterness (e.g., [10,11]), and bitter foods (e.g., grapefruit, chocolate, beer) are widely consumed and enjoyed across various cultures and cuisines. To activate bitter receptors, a ligand must fit into a binding pocket formed by a specific amino acid sequence in conserved regions of the receptor protein. Yet, the ~26 human bitter genes also show a large amount of genetic variation [12]. Changing an individual nucleotide within the protein coding sequence (a single nucleotide polymorphism; SNP) can lead to a change in the amino acid for the affected codon (i.e., a missense mutation). Numerous *TAS2R* SNPs have been shown to influence receptor activation in vitro (e.g., [13,14,15,16,17]), and these SNPs can predict bitterness, and the liking or intake of bitter compounds, foods, and beverages (e.g., [11,13,16,17,18,19,20,21,22,23,24,25]). Specifically, *TAS2R* SNPs have been associated with differential bitterness of, liking for, or intake of foods, including sweetened foods [26,27], non-nutritive sweeteners [21,28], refined cereals [29], alcohol [11,19,30,31,32,33], and vegetables [20,33,34,35,36,37]. Not all reports agree (e.g., [38,39,40,41]), but on balance, systematic reviews support a link between *TAS2R* variation and food preferences [42,43].

Variation in the ability to taste bitterness was first observed for phenylthiocarbamide (PTC) by Fox in the early 1930s [44,45], and attempts to link such individual differences to food preferences and dietary habits date back to the early 1960s [46,47,48]. Individuals with an ability to sense bitterness at low concentrations of PTC were identified as ‘tasters’, while those perceiving no taste were considered ‘nontasters’. Originally, it was hypothesized this trait was the result of a simple Mendelian trait [49,50], although modern work considers it a quantitative trait [51,52]. Following the advent of the human genome project, Kim and colleagues [12,22] were able to identify three single nucleotide polymorphisms (SNPs) in *TAS2R38* (chr. 7) that could explain the variation in the detection thresholds for PTC. These three SNPs, located at positions 49, 262, and 296, were shown to be in linkage disequilibrium, creating two common haplotypes. Thus, when dichotomized into groups based on thresholds, the ‘taster’ haplotype consists of Pro49, Ala262, and Val296, commonly abbreviated as PAV, while the ‘nontaster’ haplotype, Ala49, Val262, and Ile296, is abbreviated as AVI. Other rare haplotypes exist but reports on these [15,53,54,55] are largely inconclusive, due to the small sample sizes. The vast majority of AVI homozygotes show greatly elevated detection thresholds [56], but it is notable that some do not [53,55]. Typically, heterozygotes have lower thresholds (i.e., greater sensitivity), similar to those seen for PAV homozygotes (e.g., Figure 1 in [53]). Critically, detection thresholds are not the only taste phenotype that has been measured for bitterness, as many reports have also shown large differences in suprathreshold responses (e.g., [20,53,57,58]). Thus, these two distinct taste phenotypes—detection thresholds and rated intensity—are related but not redundant [53,59]. Here, we focus on suprathreshold intensities, not detection thresholds.

Definitionally, SNPs are variants in nucleotide sequence that may or may not result in amino acid changes in various regions of the receptor. Here, for convenience, we define a functional SNP as a polymorphism that has been shown to explain perceptual variation in vivo; critically, however, a functional SNP may not be the causal SNP that directly affects receptor binding or activation. That is, a functional SNP in vivo may act as a proxy for a mechanistically causal SNP due to linkage disequilibrium with the causal SNP. Specifically, a causal SNP could be located in different regions of the receptor, the extracellular or intracellular loops or the transmembrane domains (TM1-7), or even on another nearby gene (i.e., a long-range haplotype). For example, multiple early gene association studies suggested the Arg299Cys SNP (rs10772420) in *TAS2R19* was functional [11,24], but subsequent work strongly suggested that the causal SNP was actually located in a nearby gene, *TAS2R31* [60]. Returning to *TAS2R38*, the dominant causal SNPs appear to be at amino acid positions 49 (intracellular loop 1) and 262 (TM6). Specifically, Bufe and colleagues [15] demonstrated the functional importance of each position by expressing the *TAS2R38* variants AVI, AVV, PAV, and PVI in HEK293 cells. They found PAV and PVI had similar responses to PROP and PTC, whereas AVI and AVV exhibited no response. This suggests the amino acids located at position 49 and 262 are responsible for TAS2R38 receptor function [15]. For *TAS2R9*, Dotson et al. [14] reported that an allele at amino acid position 187, located in the TM5 domain, was critical for activation in vitro. SNPs in the extracellular loops of *TAS2R43* and *TAS2R30* may alter function by interfering with the binding pocket, while variation in the intracellular loops may interfere with signaling pathways [16]. 

Multiple studies have shown PTC and PROP activate TAS2R38 in vitro [6,15,54,55]. Notably, there is some evidence that PROP also activates TAS2R4 [61], with the caveat that the concentration of PROP used (10mM) was well above the human detection threshold and that TAS2R4 would not be activated at the concentrations typically presented when determining the threshold sensitivity or suprathreshold response [62]. Subsequent studies have found evidence that TAS2R4 responds to other bitter compounds in vitro including denatonium benzoate, peptides, and (-)-epicatechin [6,61,63,64,65]. 

Ueda and colleagues identified multiple polymorphisms in the *TAS2R4* sequence at nucleotide positions 20 (T or C), 221 (C or T), 286 (G or C), and 512 (G or A). These SNPs code for the Phe7Ser, Thr74Met, Val96Leu, and Ser171Asn alleles. Subsequently, Hayes et al. [11] observed strong linkage disequilibrium (LD) across four SNPs in *TAS2R3, -4*, and *-5,* at nucleotide position 45 (T or C) in *TAS2R3*, position 286 (G or C) in *TAS2R4*, and position 55 (A or G) and position 77 (G or T) in *TAS2R5*. Notably, the TAS2R3/4/5 diplotype explained the perceived bitterness of sampled instant espresso coffee, with individuals with one or two copies of the TGAG haplotype experiencing greater bitterness than CCGT/CCGT individuals. Elsewhere, Risso et al. showed the rs2234001 SNP in *TAS2R4* explains the variable bitterness of stevioside [66], a natural compound found in the stevia plant. Collectively, these data suggest that the activation of TAS2R4 that varies with the *TAS2R4* genotype could potentially explain the additional variation in the psychophysical response to PROP in humans.

Here, we extend prior work by showing that three SNPs in *TAS2R4* explain the phenotypic variation in the bitterness of PROP in vivo and do so independently from the *TAS2R38* genotype. Separately, we also show PROP is a weak TAS2R4 agonist in vitro at concentrations relevant to human taste perception. 

## 2. Materials and Methods

### 2.1. Overview

Data presented here were part of a large laboratory-based study with up to four test sessions, each at least one week apart. Data collected on the first day of testing are reported here; sessions 2, 3, and 4 are described elsewhere [21,67]. At the beginning of session 1, the entire study was explained to participants and written informed consent was obtained (details below). The first task was completion of a food preference questionnaire. Next, anthropometrics and salivary DNA samples were collected, followed by digital microscopy of the anterior tongue. Verbal orientation to the intensity scale was given next, including rating 15 imagined or remembered sensations [68] as a warmup task. Participants then sampled six tastants and irritants, rating them for multiple qualities. The last task in session 1 was to complete a standard propylthiouracil phenotyping protocol (details below). After the session ended, they were emailed an online survey with multiple personality measures. Total time in the laboratory for session 1 was ~1 h, and all data were collected one-on-one with project staff.

### 2.2. Participants

A prescreen was completed by each participant before the first session to ensure that they met the study’s qualification criteria. Eligibility criteria included age between 18–45 years old; not pregnant or breastfeeding; non-smoker (had not smoked in the last 30 days); no known defects of smell or taste; no lip, cheek, or tongue piercings; no history of any condition involving chronic pain; not currently taking any prescription pain medication; no reported history of choking or difficulty swallowing; and no history of thyroid disease. Participants also needed to be willing to provide a DNA sample via saliva. 

Here, we report data from 243 participants (97 men) with a mean age of 25.8 ± 0.46. Race and ethnicity were self-reported using relevant guidelines provided by the United States 1997 OMB Directive 15 guidelines; a majority of the participants were of European ancestry (*n* = 171), with others reporting Asian (*n* = 33) or African American (*n* = 6) ancestry. 

### 2.3. Ethics Statement 

Written informed consent was obtained from all participants. All procedures were approved by the Pennsylvania State University Institutional Review Board (protocol number #33176), in accordance with the Helsinki Declaration of 1975.

### 2.4. Food Preference, Anthropometrics, and Tongue Microscopy 

Following written consent, participants were given a brief set of verbal instructions on scale use before completing a 63-item hedonic survey of foods, beverages, and non-food items using an unstructured generalized hedonic scale. Anthropometric measurements (height, weight, percent body fat, and resting blood pressure) were also taken. Digital still photographs of the anterior tongue were also obtained. These data are not used here and are reported elsewhere. 

### 2.5. Psychophysical Scaling of Oral and Non-Oral Stimuli

Perceived intensities of suprathreshold stimuli were measured using a generalized Labeled Magnitude Scale (gLMS) [69]. All participants were given a verbal explanation of the scale, and then practiced using the scale, which included making ratings of 15 remembered or imagined sensations [68]. All psychophysical and hedonic data were collected using Compusense five, version 5.2 (Compusense, Inc., Guelph, ON, Canada).

Participants rated a standard assessment battery of 6-n-propthiouracil (PROP), sodium chloride (salt), and 1-kHz tones, as described previously (e.g., [11,19]). Five levels of salt and PROP were rated in duplicate, and blocks of 5 tones were repeated five times (25 in total). Stimuli were presented randomly within a block, and blocks were presented in a fixed order: 5 tones, 5 salt solutions, 5 tones, 5 salt solutions, 5 tones, 5 PROP solutions, 5 tones, 5 PROP solutions, and 5 tones. Solutions were prepared in half log steps (3.2, 1, 0.32, 0.1, 0.032 mM for PROP, and 1, 0.32, 0.1, 0.032, and 0.01 M for salt). The 1-kHz tones were presented using a calibrated Maico MA39 audiometer that had been modified to play the tones in both ears simultaneously; 1-kHz tones ranged from 50 to 90 dB in 10 dB steps. PROP and salt concentrations were prepared with USP-grade 6-n-propylthiouracil (Sigma, St Louis, MO, USA) and kosher salt in reverse osmosis (RO) water. Between each sample, participants rinsed with RO water, waiting a minimum of 30s before next sample, longer if the sensation was lingering. Overall intensity ratings for PROP were used as continuous variables rather than being binned into trichotomous groups (see [70] for rationale).

### 2.6. Participant Genotyping

DNA was collected from saliva, using Oragene collection kits according to manufacturer instructions (DNA Genotek Inc., Ottawa ON, Canada). SNPs (single nucleotide polymorphisms) in *TAS2R4* (chr 7: rs2233998 (Phe7Ser), rs2234001 (Val96Leu), rs2234002 (Ser171Asn)) and *TAS2R38* (chr 7: rs713598 (Ala49Pro), rs1726866 (Val262Ala), rs10246939 (Ile296Val)) were determined using Sequenom MassARRAY technology (Sequenom, San Diego, CA, USA) and taqman. Primers were purchased from Integrated DNA Technologies (Coralville, IA, USA). Genotypes were assigned automatically via MassARRAY software (Sequenom) and inspected by two technicians. To ensure accuracy, 15% of the samples were randomly selected and subjected to a secondary analysis. These SNP genotype frequencies did not vary from Hardy–Weinberg equilibrium: *TAS2R4* (rs2233998 (*p* = 0.42), rs2234001 (*p* = 0.74), rs2234002 (*p* = 0.78)) and *TAS2R38* (rs10246939 (*p* = 0.33), rs1726866 (*p* = 0.20), rs713598 (*p* = 0.17)).

### 2.7. In Vitro Functional Calcium Imaging Analyses

Two cDNA clones corresponding to the most frequently occurring TAS2R4 haplotype, CCA, and the second most observed haplotype, TGG, were cloned by PCR from genomic DNA and inserted into the vector pEAK10, which was modified to result in the addition of an amino terminal sst3 export tag and a carboxyl terminal addition of a herpes simplex virus glycoprotein D epitope (hsv-tag) to the coding region of the receptor [65]. The amino acid sequence of haplotype CCA exhibits a serine in position 7, a leucine residue in position 96, and an asparagine residue in position 171, whereas haplotype TGG shows phenylalanine, valine, and serine, respectively, in the corresponding positions. Accordingly, S_7_L_96_N_171_ (SLN) corresponds to the CCA haplotype, while F_7_V_96_S_171_ (FVS) corresponds to the TGG haplotype. 

HEK 293T-cells stably expressing the chimeric G protein, Gα16-Gust44, were grown in 96-well plates and transiently transfected with TAS2R4 cDNA using Lipofectamine 2000 as described before [65]. For negative controls, the empty pEAK10 vector was used (=mock). After transient transfection, the cells were allowed to express the receptor for ~24 h, before they were subjected to calcium imaging analyses. The cells were loaded with the calcium-sensitive dye Fluo-4 AM in the presence of 2.5 mM probenecid. After washing the cells with C1 buffer (130 mM NaCl, 5 mM KCl, 2 mM CaCl_2_, 10 mM glucose, 10 mM Hepes; pH 7.4), the plates were transferred into a fluorometric imaging plate reader (FLIPR Tetra, Molecular Devices, San Jose, CA, USA) and serial dilutions of 6-n-propylthiouracil in C1 buffer were automatically applied to the cells. Changes in intracellular calcium ions were monitored at a wavelength of 510 nm (488 nm excitation wavelength). Data of at least two independent experiments performed in triplicate were obtained and used for the calculation of dose–response relations. After subtraction of fluorescence changes observed in mock-transfected cells and normalization to background fluorescence, the average signal amplitudes were plotted against the logarithmic PROP concentrations using the function f(x) = [a − d/[1 + (x/EC_50_)^nH^] + d], with a = maximum, d = minimum, x = substance concentration, EC_50_ = half-maximal effective concentration, nH = −hillslope. Graphs were generated using SigmaPlot 12 (San Jose, CA, USA).

### 2.8. Statistical Analysis

Psychophysical data were analyzed using SAS 9.2 (SAS Institute, Cary, NC, USA). For individual gene analyses, analysis of variance (ANOVA) was performed via *proc mixed*. Post hoc comparisons were made via the Tukey–Kramer method. Association between polymorphisms was measured using r-squared values generated using Haploview. For *TAS2R38* and *TAS2R4*, haplotypes were determined using PHASE and individuals with probabilities less than 0.8 were relabeled as missing for that haplotype, as individuals that had at least one complete haplotype could be used in individual haplotype analyses. Relative fluorescence changes in cells transfected with TAS2R4 constructs and negative (mock) control monitored for each PROP concentration were compared by ANOVA followed by a contrast test with an alpha risk level adjusted by Bonferroni multiple testing correction using IBM SPSS Statistics for Windows, version 20.0 (Armonk, NY, USA).

## 3. Results

### 3.1. TAS2R4 and TAS2R38 Haplotypes

**Figure 1:**
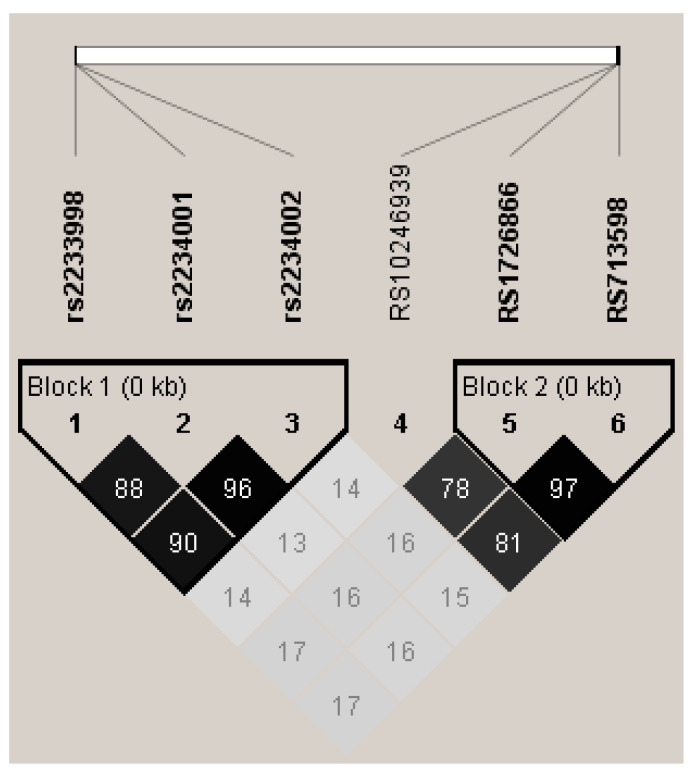
LD plot generated by Haploview with R^2^ values representing values and shading, consisting of 8 SNPs from 4 different *TAS2Rs* on chromosome 7. Haplotype blocks were defined by solid spine of LD. Block 1 shows strong LD with 3 SNPs from TAS2R4. Block 2 shows association between two SNPs in *TAS2R38* (A49P, V262A, and I296V).

In our cohort, we genotyped individuals for three SNPs in *TAS2R38* (Table 1) as well as three SNPs in *TAS2R4* (Table 2: F7S, V96L, and S171N). These SNPs are all located in a small region of chromosome 7. In our sample, we saw strong linkage disequilibrium between the three SNPs in *TAS2R4*, forming a haplotype based on solid spline LD (Figure 1). Additionally, three alleles, A49P, V262A, and I296V, in *TAS2R38* also exhibited linkage with each other, consistent with previous reports (e.g., [22]). There was minimal evidence of an association between the individual SNPs in *TAS2R4* and *TAS2R38*. This analysis was performed with the total cohort (*n* = 243). To ensure that ancestry did not alter the associations reported here, a secondary analysis was performed with only individuals who reported Caucasian ancestry (*n* = 171). In this secondary analysis, the patterns of association did not change for the *TAS2R4* and *TAS2R38* haplotypes when comparing the total sample or the Caucasian-only subsample; therefore, all of the subsequent analyses are reported for the total cohort. 

### 3.2. TAS2R4 Diplotype Explains Variation in PROP Bitterness

In our cohort, nine different haplotypes were observed for *TAS2R4*. The most frequently occurring haplotype, SLN, exhibited a frequency of 240, compared to the second most observed haplotype, FVS, with a frequency of 233. Other haplotypes were observed at a lower frequency (*n*): FLN (10), FLS (3), FVN, and SVS (1). As would be expected from these frequencies, the three most common diplotypes were SLN homozygotes (25.1%), FVS homozygotes (23.9%), and SLN/FVS heterozygotes (44.9%) (see Table 2). Accordingly, only individuals exhibiting these three most common *TAS2R4* diplotypes were used in the analysis between the *TAS2R4* diplotypes and PROP phenotype. 

**Figure 2:**
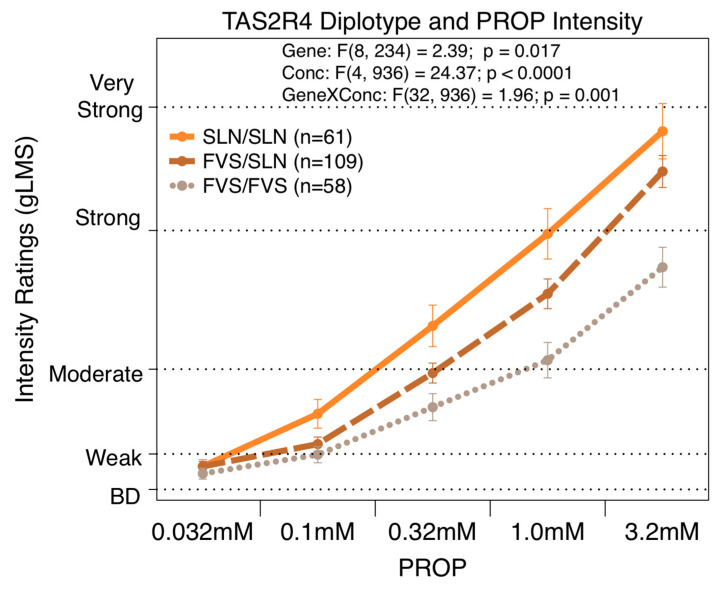
Repeated measures ANOVA reveals that reported bitterness for PROP concentrations (0.032, 0.1, 0.32, 1.0, and 3.2 mM) were significantly associated with *TAS2R4* diplotype.

The repeated measures ANOVA revealed that the *TAS2R4* diplotype was significantly associated with the gLMS intensity ratings [F(32,936) = 1.96; *p* = 0.001]) across all five concentrations of PROP, 0.032, 0.1, 0.32, 1.0, and 3.2 mM, as shown in Figure 2. The *TAS2R4* diplotype explained 7.7% of the variation for the highest concentration of PROP (3.2 mM) [F(1,206) = 17.20; *p* < 0.0001]. At the highest concentration, the FVS homozygotes rated bitterness significantly less than the SLN homozygotes or the heterozygotes, who did not differ from each other. At the second highest concentration, only the homozygous groups differed from each other.

### 3.3. TAS2R38 Explains Variation in PROP Bitterness

**Figure 3:**
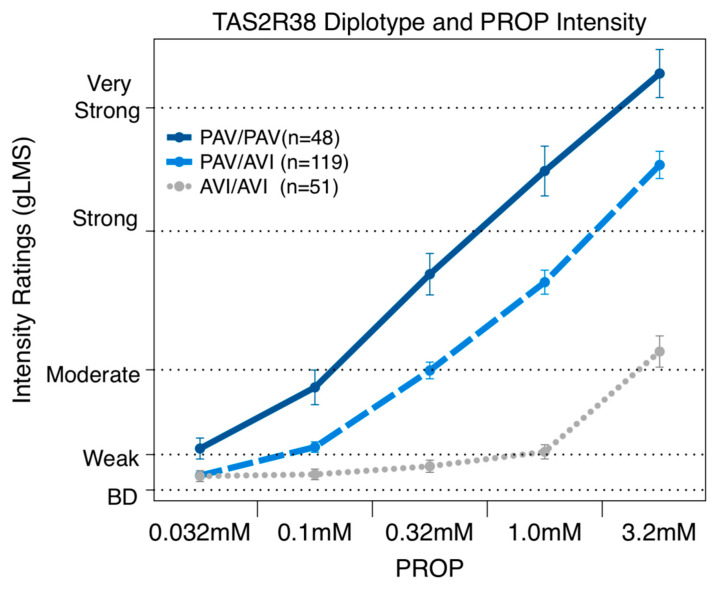
Repeated measures ANOVA reveals that reported bitterness for PROP concentrations (0.032, 0.1, 0.32, 1.0, and 3.2 mM) were significantly associated with *TAS2R38* diplotype.

Consistent with previous literature, the *TAS2R38* diplotype was a significant predictor of PROP bitterness. In our cohort, the *TAS2R38* diplotype explained 29.3% of the variation in the perceived bitterness for 3.2mM PROP [F(1,216) = 89.4 *p* < 0.0001]. The repeated measures ANOVA across all five concentrations (Figure 3) revealed that the *TAS2R38* diplotype was a significant predictor [F(8,860) = 33.96 *p* < 0.0001]. At the three highest concentrations, all three groups were significantly different from each other, as expected from prior work.

### 3.4. Combined Effects of TAS2R38 and TAS2R4 Diplotypes on Variation in PROP Bitterness

To determine the combined effects of the *TAS2R4* and *TAS2R38* diplotypes on the PROP ratings, all 207 individuals with common genotypes for both were assigned a polygenic bitterness score. The scores were assigned based on the number of higher-functioning alleles (e.g., 0, 1 or 2) for each receptor (similar to [54]), giving a minimum score of 0 (i.e., AVI/AVI and FVS/FVS individuals) and a maximum score of 4 (i.e., PAV/PAV and SLN/SLN individuals). Additional details on these scores are shown in Table 3.

The ANOVA revealed that the polygenic bitterness score based on the presumed functional *TAS2R4* and *TAS2R38* diplotypes significantly explained the variation in the bitterness ratings [F(4,202) = 15.35, *p* < 0.0001)]. The mean ratings for the 26 individuals homozygous for both of the low-functioning haplotypes (dual AVI and FVS homozygotes) had a mean rating of 18.4 (±3.9) compared to a mean of 56.8 (±3.9) for the 27 individuals homozygous for both of the high-functioning haplotypes (dual PAV and SLN homozygotes)—a ~3.1 fold difference.

### 3.5. TAS2R4 Responds to High Concentrations of PROP In Vitro

**Figure 4:**
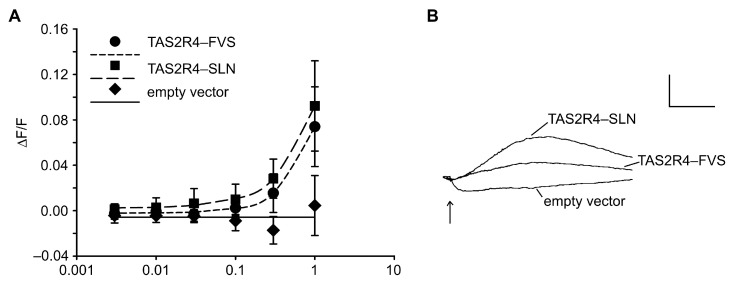
Functional calcium imaging analyses demonstrate PROP responsiveness of TAS2R4 variants. (**A**) Dose–response relationships of TAS2R4 haplotypes CCA (TAS2R4-SLN) and TGG (TAS2R4-FVS). Y-axis, relative change in fluorescence (ΔF/F), x-axis log PROP concentration in mM. (**B**) Calcium traces elicited by 1mM PROP on cells transfected with TAS2R4-SLN, TAS2R4-FVS, or empty vector. The arrow indicates the time point of stimulus application. Scale: y-axis, 100 relative fluorescence units; x-axis, 2 min.

Given the association between the *TAS2R4* genotype and PROP intensity ratings seen here, as well as the conflicting evidence in the literature concerning TAS2R4 as a low-affinity PROP receptor, we re-evaluated TAS2R4 responsiveness to PROP via functional heterologous expression assays. Accordingly, we transiently transfected HEK 293T-Gα16gust44 cells with cDNA constructs corresponding to the frequent haplotypes TAS2R4-SLN and TAS2R4-FVS. The stimulation of the transfected cells with increasing concentrations of PROP confirmed that the two TAS2R4 variants responded to the compound with an elevation in the intracellular calcium levels (Figure 4). At a concentration of 100 µM PROP, both variants exhibited significantly higher increases in fluorescence signals than the cells expressing the empty vector (negative control). At higher PROP concentrations, we observed a dose-dependent increase in the signal amplitudes for the cells transfected with the constructs confirming TAS2R4 as a PROP receptor. The pronounced receptor-independent signals occurring at concentrations higher than 1 mM PROP prevented us from monitoring the fluorescent changes at higher concentrations. Comparing the PROP-induced activation among the two TAS2R4 constructs, we observed only a slightly elevated responsiveness of TAS2R4-SLN with respect to TAS2R4-FVS, which did not reach statistical significance. Nonetheless, the effect was in the same direction as would be expected from the in vivo data, and the TAS2R4-SLN signals were consistently higher than the TAS2R4-FVS signals. 

## 4. Discussion

Through use of in vitro testing in functional expression systems, ligands have been identified for most of the 26 known human bitter taste receptors. In 2001, Chandrashekar [61] identified TAS2R4 as a potential receptor for PROP; however, subsequent work failed to replicate this finding [6]. Here, we show that PROP activates TAS2R4 in vitro. Previously, three polymorphisms in *TAS2R38* have been shown to influence receptor activation in vitro and perception in vivo for PTC and [22] and PROP [15,53,54,55].

If TAS2R38 were the sole receptor for PROP, and the AVI variant were nonfunctional (see [71]), then we would expect that the AVI homozygotes would not perceive any bitterness from PROP. However, the present data, as well as prior reports [15,19,53,54,72], suggest that some AVI homozygous individuals report bitterness at higher PROP concentrations (see Figure 3), suggesting PROP may activate additional receptors at higher concentrations. 

To test this hypothesis, we considered TAS2R4 as a candidate second receptor, since Chandrashekar and colleagues reported TAS2R4 is activated by PROP [61]. Previously, in an early pilot study that predated the identification of the functional TAS2R38 polymorphisms, Reed and colleagues [73] concluded that the *TAS2R3, TAS2R4,* and *TAS2R5* (chromosome 7) genotypes did not differ between the PROP-insensitive and -sensitive individuals. Eighteen individuals were genotyped for three SNPs in *TAS2R3* and *TAS2R4* and one SNP in *TAS2R5*. However, this unpublished abstract does not state how the PROP phenotype was determined, and it seems likely this null finding may be a false negative due to its insufficient power with just 18 participants. Based on other work focusing on coffee bitterness, we had reason to believe *TAS2R4* might contain functional SNPs, given other data associating such SNPs with differential bitterness from coffee [11] and stevia extracts [66].

Here, we confirm that PROP activates TAS2R4 in vitro. Further, we show that polymorphisms in *TAS2R4* associate with the variation in the perceived bitterness of PROP in vivo. The response of TAS2R38 to 1uM PROP in a functional expression system [54] has a similar response amplitude as TAS2R4. Still, the differential PROP response between the two TAS2R4 variants (SLN and FVS) did not reach significance in vitro. This suggests that the causal SNP altering the function of TAS2R4 may not be one of the SNPs in our analysis. However, because the amino acids at positions 7, 96, and 171 appear to explain the variation in PROP bitterness in vivo and TAS2R4 is activated by PROP in vitro, this suggests an unmeasured causal SNP may be in LD with the *TAS2R4* haplotype described here. Additionally, the TAS2R4-allele-associated differences in the PROP responses may be due to differences in receptor expression (i.e., mRNA levels), as is known to occur for TAS2R38 [74]. As for *TAS2R38*, our data are highly consistent with prior reports, explaining ~30% of the variation in the bitterness ratings of PROP here. Initially, we had hypothesized that the *TAS2R4* SNPs would explain the additional variance in the PROP bitterness in the AVI/AVI homozygotes. However, a model testing the effects of the *TAS2R38* diplotype and *TAS2R4* diplotype simultaneously did not show evidence to support this. The simplest explanation for this would be a lack of power for such a subgroup analysis (i.e., the AVI/AVI and SLN/SLN group only has four individuals). Alternatively, however, these two genes are in a similar region of chromosome 7, so it is possible their alleles are partially coinherited despite the absence of strong linkage disequilibrium in Figure 1. Such a relationship might be expected if they are under the same selective pressure. Accordingly, further work with larger cohorts is warranted to see if *TAS2R4* SNPs might explain the phenotypic functional recovery observed in some AVI homozygotes but not others. 

While the genotypes for both *TAS2R4* and *TAS2R38* each explain a significant amount of variation in the bitterness response to PROP, the *TAS2R38* effects are predominate, explaining 29.3% of the phenotypic variance, as compared to 7.7% for the *TAS2R4* diplotype at the highest concentration. Still, the present data indicate that the polymorphisms unrelated to the well-known SNPs in *TAS2R38* also explain the variation in the suprathreshold bitterness of PROP. These data show that when considering the genetic influences on a phenotype, it is important to take into account that there may be additional receptors and polymorphisms that influence taste perception. Specifically, the data reported here suggest that the widely studied *TAS2R38* polymorphisms are not the only TAS2R SNPs responsible for the variation in the PROP bitterness ratings. When considering PROP phenotypes or measuring the *TAS2R38* genotype, it is not necessary to determine the *TAS2R4* genotype; however, it is important to keep in mind that TAS2R38 is not the sole receptor for propylthiouracil. Other factors likely influence PROP perception, as *TAS2R38* haplotypes only explain a portion of the PROP variability [22]. Factors that have been previously associated with variation in PROP bitterness include mRNA expression [74] and fungiform papillae density [19,53,75,76]. Regarding food choice and food intake, a second receptor that recovers function for thiourea compounds in some AVI homozygotes may potentially explain the conflicting reports in the literature on associations between *TAS2R38* diplotypes and vegetable intake (cf. [20,41]). That is, some AVI homozygotes may behave more like heterozygotes or PAV homozygotes in terms of diet if they also have a *TAS2R4* diplotype that makes them sensitive to vegetable bitterness. 

## 5. Conclusions

In sum, the *TAS2R38* diplotype has consistently and repeatedly been shown to predict PROP bitterness in vivo, suggesting PAV homozygotes and heterozygotes have a functional receptor that causes them to perceive PROP as bitter. Further, it is widely assumed that AVI homozygotes have some degree of altered receptor function, as other data indicate that the TAS2R38-AVI variant is not activated by PROP or PTC in vitro. Despite that, many research teams have repeatedly demonstrated that many (but not all) AVI homozygotes still experience and report mild bitterness of concentrated PROP. The present data strongly implicate TAS2R4 as a functional PROP receptor, in agreement with prior speculation on the existence of a second lower affinity PROP receptor [53]. Polymorphisms in *TAS2R4* have previously been linked to the differential bitterness of coffee and stevia plant extracts, but it remains unknown whether these same polymorphisms are predictive of dietary intake, especially in relation to vegetables and/or alcohol.

## Figures and Tables

**Table 1:** Summary demographics and diplotypes of study participants.

Diplotype	*n* (%)	Asian	African American	White/Caucasian	Male/Female
PAV/PAV	48 (19.8%)	14	3	24	24/24
PAV/AVI	119 (49.0%)	9	2	90	46/73
AVI/AVI	51 (21.0%)	9	-	37	17/34
Rare	25 (10.3%)	1	1	20	10/15
Total	243	33	6	171	97/148

**Table 2:** *TAS2R4* SNPs and counts in the study participants.

rs2233998	rs2234001	rs2234002	Frequency (%)
S/F	L/V	N/S	109 (44.9%)
S/S	L/L	N/N	61 (25.1%)
F/S	V/V	S/S	58 (23.9%)

**Table 3:** Polygenic bitterness score based on *TAS2R38* and *TAS2R4* diplotypes.

Score	*n*	Contributing Groups (*n*)
0	26	AVI/AVI + FVS/FVS
1	39	AVI/AVI + FVS/SLN (16) PAV/AVI + FVS/FVS (23)
2	79	AVI/AVI + SLN/SLN (4) PAV/AVI + FVS/SLN (71) PAV/PAV + FVS/FVS (4)
3	36	PAV/AVI + SLN/SLN (22) PAV/PAV + FVS/SLN (14)
4	27	PAV/PAV + SLN/SLN

## Data Availability

Data are not available, as the consent documents did not ask participants about resharing these data.
